# Supplementary material for: Dynamic transcriptomic profiles of zebrafish gills in response to zinc supplementation
Source: BMC Genomics. 2010 Oct 11;11:553. doi: 10.1186/1471-2164-11-553 (PMC3091702; doi:10.1186/1471-2164-11-553)
Supplement: Additional file 2 — Interactive Direct Interaction Network representing the molecular interactions between zinc, copper, iron, calcium and proteins encoded by transcripts changed by zinc supplementation. Mini web-site containing index.html and hyperlinked pages in subdirectory describing a Direct Interaction Network automatically generated based on curated interactions contained within the proprietary PathwayArchitect database. Ovals represent proteins and the circles symbolize metal ions. Objects are coloured by their abundance in zebrafish at the time-point they were significantly different from the control is a scale from -4 fold (dark green) to +4 fold (dark red). Where significant differences were found at more than one time-point, the colour overlay shows expression at the first instance. Dark blue squares denote 'binding', and light blue squares 'expression'; green squares stand for 'regulation', green diamonds for 'metabolism', and green circles for 'promoter binding'. Arrow heads indicate directionality of the interaction where annotated. All nodes and edges can be further interrogated by selecting the relative area of the image. [file 1471-2164-11-553-S2.zip › PathwayArchitect Zn xs DIN/1484951.html]

# REGULATION:

|  |  |
| --- | --- |
| Type | REGULATION |
| Effect | Positive |


---

|  |  |
| --- | --- |
| Score | 0 |


---

|  |  |
| --- | --- |
| Reference Count | 7 |


---

|  |  |
| --- | --- |
| Mechanism | Unknown |


---

|  |  |
| --- | --- |
| Reference:0 || Sentence | "Analysis of intracellular Ca(2+) cycling in S100A1-overexpressing cardiomyocytes revealed a significant increase in cytosolic Ca(2+) transients, whereas in functional studies on saponin-permeabilized adult cardiomyocytes, the addition of S100A1 protein significantly enhanced SR Ca(2+) uptake." |
| PMID | 11717446 |
| Year | 2001 |
| Species | Human |
| Journal | Proc Natl Acad Sci U S A |
| RefScore | 1 |
| Source | PArchNLP |
  |
|  |  |


---

|  |  |
| --- | --- |
 Reference:1 || Sentence | "We therefore investigated the impact of adenoviral S100A1 overexpression on fractional shortening (FS%) and systolic Ca2+ transients in adult rat cardiomyocytes as well as of S100A1 protein on SERCA activity in skinned cell preparation." |
| PMID | 12479236 |
| Year | 2002 |
| Species | Human |
|  | Rat |
| Journal | Basic Res Cardiol |
| RefScore | 0 |
| Source | PArchNLP |
  ||  |  |


---

|  |  |
| --- | --- |
 Reference:2 || Sentence | "Thus, S100A1 enhances cardiac contractility by increasing intracellular Ca2+ fluxes at least in part due to a modulation of SERCA." |
| PMID | 12479236 |
| Year | 2002 |
| Species | Human |
|  | Rat |
| Journal | Basic Res Cardiol |
| RefScore | 2 |
| Source | PArchNLP |
  ||  |  |


---

|  |  |
| --- | --- |
 Reference:3 || Sentence | Voltage clamp experiments (20 degrees C) indicated that 0.1 microM S100A1 increased Ca(2+) transient amplitude by approximately 41% but higher or lower S100A1 concentrations had no significant effect. |
| Year | 2005 |
| PMID | 16236309 |
| Species | Human |
| Journal | J Mol Cell Cardiol |
| RefScore | 2 |
| Source | PArchNLP |
  ||  |  |


---

|  |  |
| --- | --- |
 Reference:4 || Sentence | Therefore, 0.1 microM S100A1 appeared to increase the fractional Ca(2+) release from the SR. |
| Year | 2005 |
| PMID | 16236309 |
| Species | Human |
| Journal | J Mol Cell Cardiol |
| RefScore | 1 |
| Source | PArchNLP |
  ||  |  |


---

|  |  |
| --- | --- |
 Reference:5 || Sentence | In addition, S100A1 overexpression enhanced both maximal Ca(2+)-stimulated force generation (+81%; P < 0.05) and Ca(2+)-sensitivity of EHTs (EC50% [Ca(2+)](e) mM; AdvGFP 0.33 +/- 0.04 vs. AdvS100A1 0.21 +/- 0.0022; P < 0.05). |
| Year | 2004 |
| PMID | 15079813 |
| Species | Rat |
| Journal | J Gene Med |
| RefScore | 1 |
| Source | PArchNLP |
  ||  |  |


---

|  |  |
| --- | --- |
 Reference:6 || Sentence | Extracellularly added S100A1 enhanced the Ca2+-transient amplitude in neonatal ventricular cardiomyocytes (NVCMs) through a marked decrease in intracellular diastolic Ca2+-concentrations ([Ca2+]i). |
| PMID | 15654019 |
| Year | 2005 |
| Species | Human |
|  | Rat |
| Journal | J Cell Sci |
| RefScore | 1 |
| Source | PArchNLP |
  |


---

|  |  |
| --- | --- |
